# Surface regulation enables high stability of single-crystal lithium-ion cathodes at high voltage

**DOI:** 10.1038/s41467-020-16824-2

**Published:** 2020-06-16

**Authors:** Fang Zhang, Shuaifeng Lou, Shuang Li, Zhenjiang Yu, Qingsong Liu, Alvin Dai, Chuntian Cao, Michael F. Toney, Mingyuan Ge, Xianghui Xiao, Wah-Keat Lee, Yudong Yao, Junjing Deng, Tongchao Liu, Yiping Tang, Geping Yin, Jun Lu, Dong Su, Jiajun Wang

**Affiliations:** 1 MIIT Key Laboratory of Critical Materials Technology for New Energy Conversion and Storage, School of Chemistry and Chemical Engineering, Harbin Institute of Technology, 150001 Harbin, China; 2 Center for Functional Nanomaterials, Brookhaven National Laboratory, Upton, NY 11973 USA; 3 Chemical Sciences and Engineering Division, Argonne National Laboratory, Lemont, IL 60439 USA; 4 SSRL Materials Science Division, SLAC National Accelerator Laboratory, Menlo Park, CA 94025 USA; 5 National Synchrotron Light Source II, Brookhaven National Laboratory, Upton, NY 11973 USA; 6 Advanced Photon Source, Argonne National Laboratory, Lemont, IL 60439 USA; 7 College of Material Science and Engineering, Zhejiang University of Technology, 310014 Hangzhou, China; 8 Beijing National Laboratory for Condensed Matter Physics, Institute of Physics, Chinese Academy of Sciences, 100190 Beijing, China

**Keywords:** Batteries, Electrochemistry

## Abstract

Single-crystal cathode materials for lithium-ion batteries have attracted increasing interest in providing greater capacity retention than their polycrystalline counterparts. However, after being cycled at high voltages, these single-crystal materials exhibit severe structural instability and capacity fade. Understanding how the surface structural changes determine the performance degradation over cycling is crucial, but remains elusive. Here, we investigate the correlation of the surface structure, internal strain, and capacity deterioration by using *operando* X-ray spectroscopy imaging and nano-tomography. We directly observe a close correlation between surface chemistry and phase distribution from homogeneity to heterogeneity, which induces heterogeneous internal strain within the particle and the resulting structural/performance degradation during cycling. We also discover that surface chemistry can significantly enhance the cyclic performance. Our modified process effectively regulates the performance fade issue of single-crystal cathode and provides new insights for improved design of high-capacity battery materials.

## Abstract (web summary)

Performance improvement of cathode materials represents one of the most critical technological challenges for lithium-ion batteries. Here the authors show a close correlation between surface chemistry and phase distribution from homogeneity to heterogeneity, and its impact on the cyclic performance.

## Introduction

Specific capacity improvement has been reported in high Ni content lithium nickel cobalt manganese oxide (NCM) electrodes for lithium-ion batteries (LIBs) charged at high voltage. However, the increased voltage cutoffs also aggravate material decomposition and impede the battery performance^1–3^. It is commonly accepted that these transitions from layered to spinel or rock-salt phases, and migration/segregation of transition metals (TMs) induce structural reconstruction that facilitates capacity fade^4–6^. During delithiation, the layered oxide material may transform into a spinel-type phase and then to a completely disordered rock salt-type structure, which is believed to inhibit the diffusion of lithium ions. In addition, the dissolution, migration, and segregation of TMs on the surface further deteriorate battery performance^7–9^. Although some traditional methods doping and coating strategies have been reported to inhibit cation mixing and suppress interfacial reactions, the excessive coating layer (>20 nm) and unregulated doping strategy may hinder the migration of Li^+^, resulting in poor rate capability^10,11^. Therefore doping strategies would consequently decrease specific capacity. Another issue was recently raised that uneven stresses, observed in cycles driven at high voltage, induced intragranular cracks in polycrystalline, which exacerbated structural collapse and capacity loss in Ni-rich NCM^12–14^. To solve the deficiencies caused by different reasons, creative strategies are necessary to improve structural stability in NCM particles^15^. Ideal methods should be able to simultaneously tune structural and morphological characteristics to restrict both structural failure and intragranular cracks^13,16,17^.

Single crystalline NCM with only one grain for one particle (grain sizes of 2–5 μm) have attracted increasing attention for the cathode of LIBs due to their superior capacity retention during long cycle times, which exceeds conventional polycrystalline battery particles^18,19^. It was believed that such good capacity retention originated from their high levels of structural stability^19–22^. First, the cracking problems of NCM can be potentially suppressed because of the exhibited intrinsic integrity and continuous conductive networks in single-crystal particles^23^. Second, without grain boundaries in particles, single-crystal electrodes were theorized to provide increased oxygen loss resistance and sound structural stability in interactions with electrolyte when compared with polycrystalline materials^24^. Although single-crystal NCM electrodes can eliminate the grain boundary resistance, degradations from NCM electrodes itself still occur and intrinsically hinder the further improvement of practical applications. Few studies elucidated the deterioration mechanism of single-crystalline NCM at high voltage. Understanding the structure-performance correlations of NCM single crystal can not only solve the above issue but also provide fundamental insights into the degradation mechanism of polycrystalline NCM electrodes, with a clarification on the role of the grain boundaries.

In this work, we seek to understand underlying recession mechanisms in single-crystalline battery materials with using *operando* synchrotron X-ray spectroscopic microscopy and X-ray nanotomography. Ni-rich single-crystal LiNi_0.6_Co_0.2_Mn_0.2_O_2_ (NCM622) was selected as a model electrode because it exhibits a high specific capacity of over 220 mAh g^−1^. To correlate structural/morphological changes with cycle capability, mesoscale phase distributions during long-term cycling were visualized at single-crystalline levels. Results from comprehensive testing reveal that surface physical character, such as phase transitions from homogeneity to heterogeneity during cycling, induce particle crack formations, and play a dominant role in the structural robustness of single crystals. Moreover, we discover that surface regulated approaches could mitigate this undesirable phase evolution in single-crystal NCM and significantly enhance cycle performance. Our gather evidence consequently elucidates the relationship between surface chemistry, phase transition, and performance retention, while providing new guidelines for the rational design of high performance, stable, layered cathode materials^25–27^.

## Results and discussion

### Performance decay of single crystals at high-voltage cycling

**Fig. 1:**
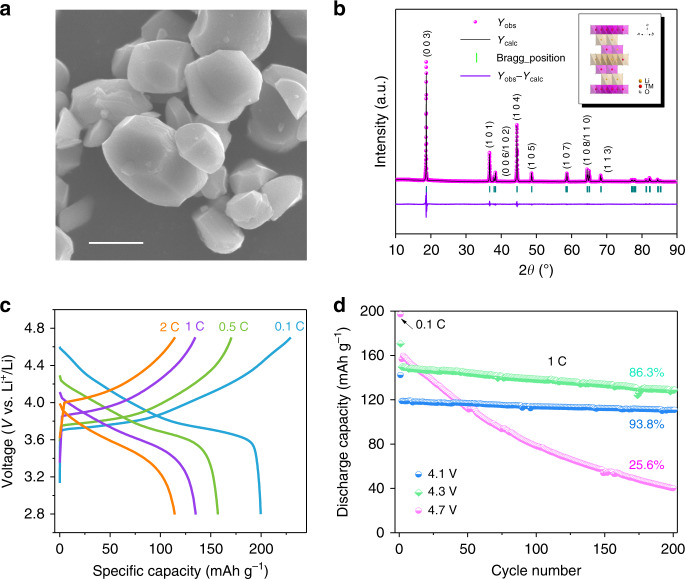
Performance decay of single-crystal batteries at high-voltage cycling. **a** SEM images of NCM. Scale bars 2 μm. **b** Rietveld refinement results for NCM samples (the illustration is the crystal structure of NCM). **c** Charge–discharge profiles of NCM at different rates. **d** Cycling performances at 1 C (1 C = 270 mA g^−1^) under different cut-off voltages (4.1, 4.3, 4.7 V).

Singe-crystalline NCM particles exhibit well-defined polyhedral shapes with particle sizes of 1–5 μm (Fig. 1a and Supplementary Fig. 1). We have performed a transmission electron microscope (TEM) analysis on single particles of NCM. From the bright-field TEM images and corresponding selected area electron diffraction patterns, as shown in Supplementary Fig. 2, we concluded that these single particles are single crystal. Major X-ray diffraction (XRD) reflections in Fig. 1b can be indexed as *R*-*3m* space group (JCPD, 09-0063), which is in good agreement with the hexagonal *α*-NaFeO_2_ type crystal structure^21^. The clear splitting peaks of (006)/(012) and (018)/(110) imply a well-organized crystalline layered structure. Rietveld refinement exhibits a well-ordered layered structure with no evidence of other impurity phases. Also, the results show that the mixing of Li/Ni in the NCM is common in the synthesis of layered oxides (Supplementary Table 1). NCM single crystals showed a high specific capacity of 197.8 mAh g^−1^, which is equivalent to most reported results in NCM samples (Fig. 1c). Furthermore, we carried out electrochemical stability measurement with different upper cutoff voltages (4.1, 4.3, and 4.7 V) (Fig. 1d and Supplementary Fig. 3). When cut-off voltages were below 4.3 V, single-crystal NCM exhibited significantly increased cycling performance. However, at a high charge cutoff voltage of 4.7 V, single-crystal NCM displayed decreased stability with a capacity retention of only 25.6% after 200 cycles. Nevertheless, in order to increase specific capacity, battery materials must be stable at higher charge voltages. Therefore, insight into the failure mechanism of high-voltage NCM single-crystal cathodes holds great value for capacity enhancement.

### Structural analysis of cycled single-crystal material

**Fig. 2:**
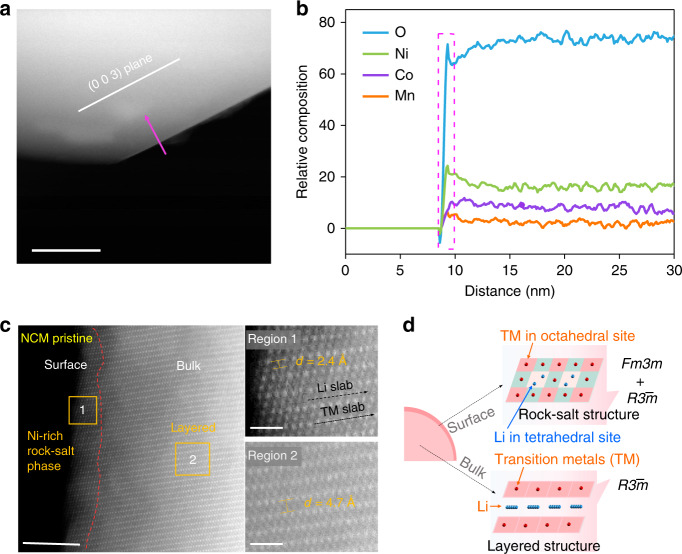
Surface analysis of pristine NCM electrodes. **a** STEM image of NCM. **b** A line scan of EELS showing the elemental profiles of the relative atomic concentration of Ni, Co, Mn on pristine NCM particle surface. **c** Atomic resolution HAADF-STEM images of pristine NCM samples. Scale bars 5 nm, region 1: 1 nm, region 2: 1 nm. **d** Schematic diagram for atomic configuration of NCM bulk and surface.

The surface chemistry of single-crystal NCM was conducted with TEM, and the line scan and mapping of the electron energy loss spectroscopy reveal the presence Ni-rich phase on the surface (Fig. 2a, b and Supplementary Fig. 4), which often corresponds to a rock-salt phase^5,28^. To further visualize this surface structure at atomic resolutions, we performed high-angle annular dark-field (HAADF)-scanning transmission electron microscopy (STEM) on pristine single-crystal NCM. The inner zone of NCM corresponds to the layered phase, while the surface region (~3 nm) shows a rock-salt phase, which confirms the presence of a thin Ni-rich rock-salt layer on the single-crystal particle surface (Fig. 2c)^29^. As for polycrystal sample, it is generally accepted that the surface configuration of this thin rock-salt layer can significantly affect the electrochemical behavior of NCM layered oxide cathode (Fig. 2d). Nevertheless, the true relationship between surface chemistry and structural stability for single crystal NCM remains unclear, which requires comprehensive studies to understand its configuration and mechanism of impact on electrochemical performance.

**Fig. 3:**
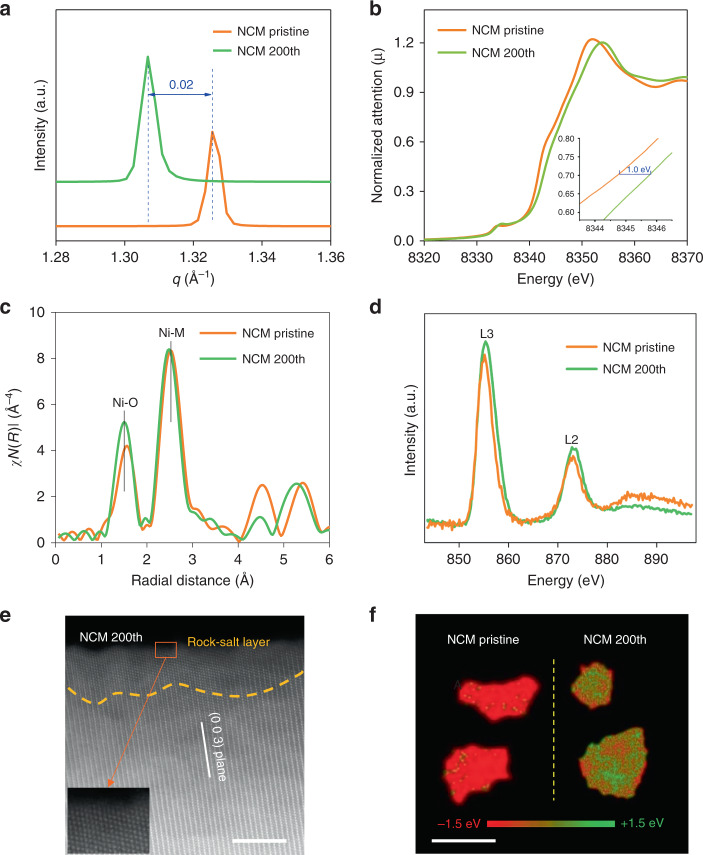
Structural analysis of pristine and cycled NCM electrodes. **a** Synchrotron XRD pattern for NCM. **b,** **c** Ni K-edge XANES and EXAFS spectra of pristine and cycled NCM crystals. **d** Soft XAS spectra of Ni L-edge for pristine and 200th cycle NCM. **e** Atomic resolution HAADF-STEM images of 200^th^ cycled NCM samples. **f** Ex-situ 2D TXM of pristine and cycled NCM. Scale bars 2 μm.

**Fig. 4:**
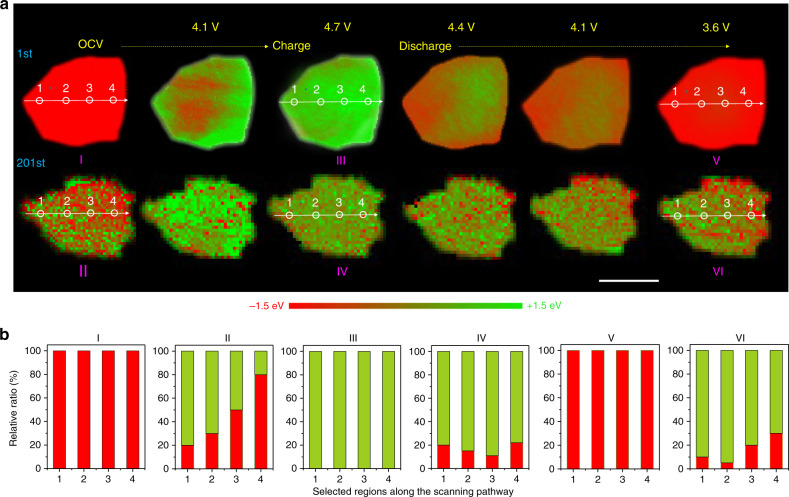
Spatial-resolved electrochemical evolution after the 1st and 201st cycle. **a** *Operando* 2D chemical phase mappings at the Ni K-edge of NCM particles during the first and 201st cycles. Scale bars 2 μm. 1st (I, pristine; III, 4.7 V; V, 3.6 V), and 201st cycle (II, pristine; IV, 4.7 V; VI, 3.6 V), **b** Quantification of the selected positions in 2D TXM-XANES phase mappings (Red represents discharge state, green represents charge state).

Electrodes harvested from cells were analyzed using various techniques to understand the structural evolution and degradation of single-crystal NCM at high potential. First, synchrotron XRD was performed on samples cycled at a high cut-off voltage (4.7 V) to understand structural transformations. As shown in Fig. 3a, the shift at the (003) peak indicates low reversibility of lithium deintercalation in single-crystal NCM after 200 cycles^23^. Fine chemical and structural information were also analyzed using hard X-ray absorption spectroscopy (Fig. 3b). The cycled single-crystal electrode exhibits a ~1.5 eV edge energy shift toward higher energy in the Ni K-edge X-ray absorption near edge structure (XANES) spectrum, which reflects an oxidation state increase for Ni in single-crystal NCM. Extended X-ray absorption fine structure (EXAFS) spectroscopy results (Fig. 3c), which are more sensitive to local chemical and structural changes, clearly show that Ni–O and Ni–M interatomic distances are shortened after electrochemical cycling. Changes in the chemical state of Ni and the atomic environment could be ascribed to the presence of unfilled lithium vacancies during lithiation^30^. Surface-sensitive soft X-ray absorption spectroscopy (XAS) measurements were also conducted to further understand the surface chemical change in single-crystalline NCM. Nickel L-edge spectra have an L_3_ (2*p*_3/2_ → 3*d*) and an L_2_ (2*p*_1/2_ → 3*d*) region due to spin–orbital coupling. The shape, energy, position, and peak intensity ratio contain information on sample valence states. A decreased L_3_/L_2_ ratio was found in the spectrum after cycling, which provides evidence of Ni elements that have undergone oxidation reactions in at the single-crystal surface (Fig. 4d)^31^ This result was also confirmed with X-ray photoelectron spectroscopy analysis (Supplementary Fig. 5). With such comprehensive X-ray analysis, we decisively conclude that serious structural degradation and surface configuration transitions occur in single-crystal NCM after high-voltage cycling (Fig. 1c).

The heterogeneity of phase distribution has been observed in polycrystalline NCM^32^. It is generally accepted that this phase heterogeneity at the chemically delithiated samples is similar to that of electrochemically charged samples in large polycrystals. However, the mapping study of phase distribution was seldom reported in single-crystal battery materials^33^. Therefore, we propose that surface configuration plays a role in the cycling capability of pristine single-crystal particles. After 200 cycles, the single crystals showed a disordered layer (~5 nm depth) on the surface of the particle as expected (Fig. 3e), which suggests that the cycled cathode exhibited extreme instability. This surface Ni-rich rock-salt phase will hinder lithium-ion transport, and we speculate that it will affect the inner chemical phase of the single-crystal particles during long-term cycling. In order to further quantitatively describe structural transformation at particle-scales, we conducted spatially resolved synchrotron transmission X-ray microscopy (TXM) to probe chemical changes in single NCM crystals after cycling (Fig. 3f). Inhomogeneous distributions of Ni^3+^ and Ni^4+^ oxidation states were found to coexist in the single-crystal particle, which indicated irreversible chemical changes to NCM after battery cycling. This inhomogeneous phase distribution may be caused by lithium-ion diffusion within single crystals, which is related to surface chemical features in single-crystal particles.

### Spatially-resolved structural evolution

In order to gain a deeper understanding of the correlation between surface chemistry and performance decay, in *operando* TXM was performed to monitor the compositional and structural evolution before and after 200 cycles^25,34^. During the initial cycle, the redox reaction occurs along all directions and phase-transition fronts propagate drastically, as visualized by the in *operando* quantitative chemical mapping in Fig. 4a. A near-complete phase transition occurs at the final charge and discharge stage, indicating highly electrochemical reversibility. Nevertheless high reversibility cannot survive after 200 cycles, which is unambiguously revealed through *operando* TXM chemical phase mapping. During the 201st charge cycle, it was found that around 50% of the phase composition could not recover to a pristine state, and a highly heterogeneous phase distribution appears in single-crystal NCM. Moreover, phase transition at the same location is highly irreversible during the 201st cycle, as evidenced in the composition analysis of Fig. 3b. Four representative regions from the bulk to the surface were selected and the corresponding content of Ni oxidation states were illustrated in Fig. 4b. The 2D histogram highlights inhomogeneous Ni oxidation state distribution within the single-crystal particle. Reaction heterogeneity and high irreversibility could be ascribed to the characteristics of surface chemicals (Ni-rich rock-salt phase) on pristine single NCM crystals, which may induce heterogeneous internal strain within the particle, and further result in structural/performance degradation, as evidenced with the chemical composition analysis of Fig. 4b and Supplementary Fig. 6.

**Fig. 5:**
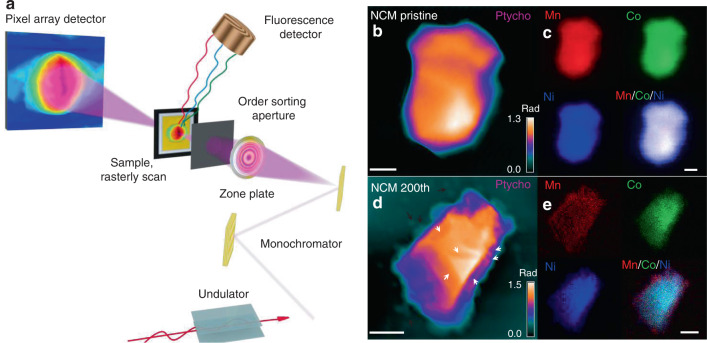
Simultaneous X-ray fluorescence and ptychography. **a** Experimental schematics. **b**–**e** X-ray fluorescence and ptychographic images of pristine and 200th cycled NCM particles to show element distribution and morphology. Scale bars: **b** 2 μm, **c** 1 μm, **d** 2 μm, **e** 1 μm.

**Fig. 6:**
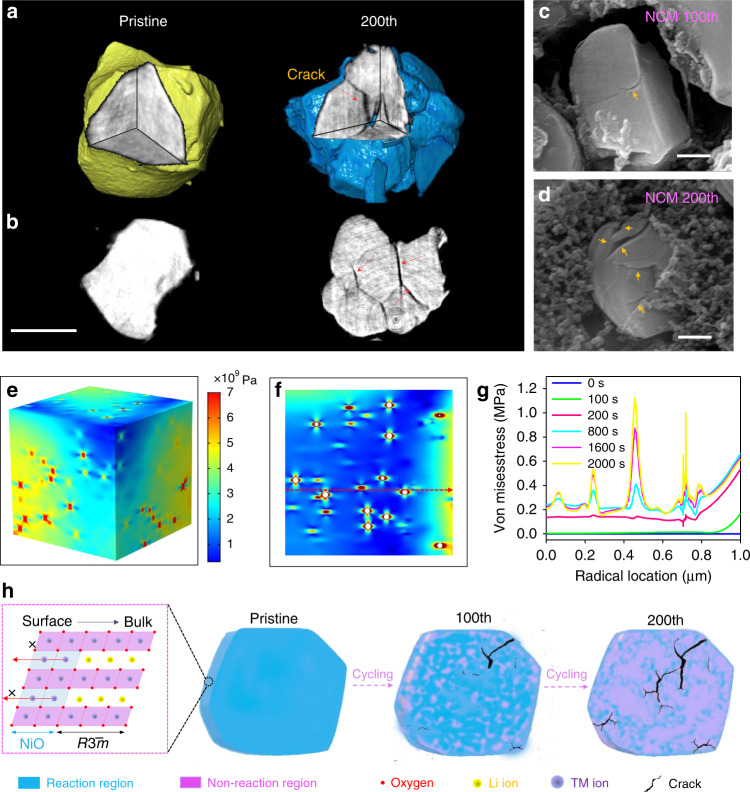
3D reconstruction and structural degradation mechanism. **a, b** X-ray nano-tomography reconstruction with volume rendering shows the morphological evolution of NCM after cycling. Scale bars 2 μm. **c**, **d** SEM images of the NCM microsphere after 100 and 200 cycles. Scale bars 500 nm. Equivalent stress within the NCM particles: **e** 3D semitransparent view. **f** 2D view of the cross profile. **g** Von Mises stress within the NCM particles along the imaginary line. **h** structural degradation mechanism in NCM.

To probe the influence of trace surface elemental rearrangement on chemical and physical functions, we sought a novel analysis tool with high elemental sensitivity. X-ray fluorescence microscopy (XFM) provides unparalleled sensitivity for trace element distribution measurements in many micrometer-thick specimens (true microscale battery particles) and facilitates significantly improved sensitivity relative to electron probes^35^. With X-ray ptychrography, an emerging method that images ultrastructures at beyond-focus-optic resolution, a combined approach with XFM and ptychography can be employed to study elemental localization within the high-resolution structural context, which aids the elucidation of phase transition mechanisms^36–38^. The fluorescence maps (Mn, Co, Ni) in Fig. 5 indicate homogeneous elemental distributions of Ni, Co, and Mn within the pristine single-crystalline particle, while Mn metal segregation and Ni deficiencies are observed within the single-crystalline particle after 200 cycles^7^. The phase images given by ptychography reveals the projected electron density distribution of the particle along the X-ray beam direction. For the pristine sample in Fig. 5b, the phase map shows a single intact crystal with several facets, which is consistent with the SEM image of pristine particles (Fig. 1a). However, ptychographic image of the particle after 200 cycles shows inhomogeneous morphology, several example locations indicated by white arrows have lower electron density, which is presumably due to the formation of cracks. In addition, small particles with weak phase (indicated by black arrows in Fig. 5c) were observed around the particles, which come from the electrolyte. In all, the projected phase image of the cycled particle is well consistent with the surface morphology given by SEM (Fig. 5d). To better visualize the internal structures and further directly correlate microstructural changes to electrochemical cycle stability, we performed synchrotron X-ray nanotomography^39^. Fig. 6a illustrates the 3D microstructure of pristine and 200th cycled single-crystalline NCM. Significant amounts of cracks in single-crystalline particles indicate that the integrity of the original microstructural morphology has been destroyed. Close examination of NCM particles after the 200th cycle reveals multiple microcracks on the particles (indicated by the arrow in Fig. 5), which are gaps between primary particles that arose from inhomogeneous diffusion during the cycling process. Figure 6b reveals the internal structural information of the split sample from the 2D projection images extracted along the *Z* vertical axis. For cycled samples, present internal cracks and fractures can be attributed to heterogeneous phase transition-induced internal strains during long-term cycling. This is consistent with results obtained from SEM (Fig. 6c, d).

It is well known that the Ni-rich rock-salt phase on the surface caused by cation mixing may inhibit lithium-ion transport, which can trigger surface-phase transformations from layered to rock-salt structures and induce inhomogeneous lithium-ion distribution^40,41^. We employed diffusion-induced stress models to understand the Electrochem-mechanical degradation mechanism and investigate stress change in single-crystal NCM^15^. Fig. 6e illustrates the heterogeneous stress distribution caused by the inhomogeneous distribution of lithium-ion concentration in single-crystal NCM particles. Such inhomogeneous lithium distribution may cause mismatched strains, which leads to high-stress concentrations near the phase interface (Fig. 6f, g). As cycles proceed, the particles will be lacerated when the fracture strength is unable to sustain the strains, which quickly induces polarization and plummets cycle performance. This mechanism is schematically illustrated in Fig. 6h. As for untreated NCM single crystals, the Ni-rich rock-salt phase of a single-crystal surface inhibits near-surface lithium-ion transport, which results in heterogeneous chemical particle distribution and causes stress generation. Deep lithium extraction/intercalations and stress release further increase internal strain and the presence of intergranular cracks, which decreases the structural robustness of NCM materials.

### Single-crystal NCM with modified surface chemistry

**Fig. 7:**
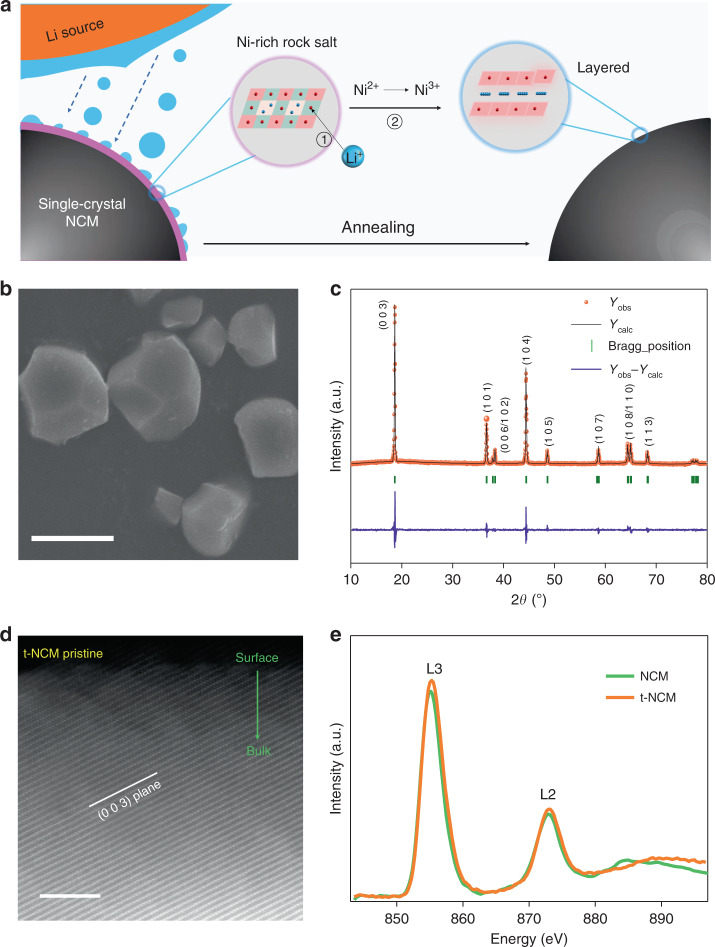
Surface structure and morphology analysis of the t-NCM. **a** Diagram of the synthesis process and mechanism for modified NCM. **b** SEM image of pristine t-NCM. **c** XRD of pristine t-NCM **d** Atomic resolution HAADF-STEM images of pristine and cycled t-NCM samples. Scale bars 5 nm. **e** Soft XAS spectra of Ni L-edge for t-NCM.

Our results reveal that the surface Ni-rich rock-salt phase plays a major role in the cycle stability of single-crystal NCM. To address this issue, efforts should be made to eliminate surface chemical reconstruction in single crystals. It is well known that lithium could redeposit into the single crystals surface lattice via oxidation of the rock-salt Ni^2+^ phase to layered Ni^3+^, which can restore the particle surface to a well-ordered lattice structure. Inspired by this finding, we attempted to modify the surface Ni-rich rock-salt phase using a feasible lithium source to replenish lattice sites during re-calcination under oxygen flow and high-temperatures (denoted as t-NCM)^42,43^. From a fundamental perspective, when the NCM is calcined at high temperature with O_2_ flow, the lithium ions from LiOH could return to the lattice. Simultaneously, Ni^2+^ in the Ni-rich rock-salt structure can be reoxidized to Ni^3+^ and restored to the layered structure (Fig. 7a). SEM images in Fig. 7b help conclude that our heat-treatment process has a negligent effect on particle morphology and size. From XRD analysis, the treated single crystals exhibit the layered *R-*3m phase which is the same as the untreated one (Fig. 7c). The intensity (003) to (104) peak ratio, which is an indicator of the degree of cation mixing in layered structures with an *R*-3m space group, increases from 1.56 to 1.79, which indicates a decrease in cation mixing after treatment^29^. (Supplementary Table 2). It could be reasoned that this vanished Ni-rich rock-salt phase may be related to the lithium source supplements during re-calcination. From synchrotron X-ray absorption analysis, no drastic variation of Ni K-edge XANES in t-NCM single-crystal samples (Supplementary Fig. 7a) is observed, which suggests the average valance state of Ni does not change after heat treatment. Noticeably, the amplitude of the Ni-O and Ni-TM peaks in Ni K-edge EXAFS spectra decreases significantly after treatment (Supplementary Fig. 7b) This further verifies decreased LiNi6 cation ordering in transition metal layers of modified NCM and is in good agreement with prior XRD analysis^44^.

**Fig. 8:**
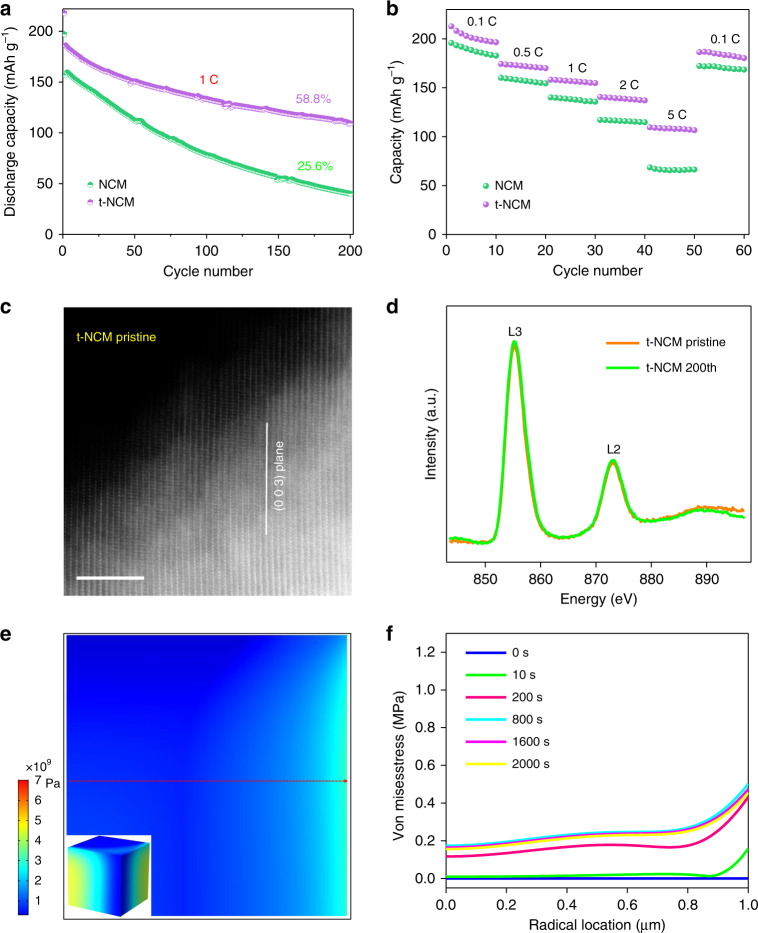
Structural analysis of improved stability in the t-NCM. **a** Cycling performance comparison plot of untreated NCM and t-NCM. **b** NCM and t-NCM rate capabilities. **c** Atomic resolution HAADF-STEM images of pristine and cycled t-NCM samples. Scale bars 5 nm. **d** Soft XAS spectra of Ni L-edge for t-NCM. Equivalent stress within the NCM particles: **e** 2D view of the cross profile. The illustration in the picture is 3D semitransparent view. **f** Von Mises stress within the NCM particles along the imaginary line.

Slight structural changes in the single crystals before and after treatment may be easily overlooked by some conventional characterization technologies. In addition, we assume this subtle change may probably occur near-surface region of single crystals as the treatment occurs in oxygen under high temperatures. HAADF-STEM study was carried out and it was found that t-NCM exhibits a layered phase on the surface, which indicated that our surface treatment can replenish the disordered surface structure (Fig. 7d). In the Ni L-edge spectra of the NCM and t-NCM samples, the positive shift of the adsorption edge t-NCM indicates the removal of Ni-rich rock-salt structure layer after treatment (Fig. 7e)^31^, which is consistent with the results obtained in Fig. 7d. We evaluated the electrochemical properties of single-crystal t-NCM. Both initial charge/discharge capacity and electrochemical reversibility were remarkably enhanced after surface chemistry treatment (details in Supplementary Fig. 8). In particular, cycle stability is increased significantly from 25.6% to 58.8% (Fig. 8a and Supplementary Fig. 9) and t-NCM also shows excellent rate performance (Fig. 8b). These improved electrochemical performances can be attributed to the disappearance of the Ni-rich rock-salt phase layer on the single-crystal surface. Even after 200 cycles, the layered structure of the modified single-crystal t-NCM is still well maintained, as shown in Fig. 8c.

X-ray technologies were further employed to understand t-NCM structural changes. Robust crystal structures with no obvious pattern change were confirmed from the XRD results of cycled 9t-NCM single crystals (Supplementary Fig. 10). Ni K-edge XANES spectra of cycled t-NCM samples display negligent edge shifts, and information on the nickel local environment (Ni-O and Ni-TM in EXAFS) also suggest high structural stability (Supplementary Fig. 11). Soft XAS was also conducted to visualize this structural change on modified single-crystal particle surfaces and to probe surface character at the single-crystal surface. The surface chemical states of these crystals can be determined by examining the fine structures of L_3, 2_ absorption edges. As revealed in Fig. 8d, the low-energy shoulder of L_3_-edge and the L_3_/L_2_ intensity ratio show negligible change in t-NCM before and after 200 cycles, which is indicative of high surface-chemical stability. In addition, t-NCM crystals retain homogeneous phase distribution across single crystals, as illustrated in X-ray spectroscopy imaging via TXM (Supplementary Fig. 12). These cumulative results highlight the crucial influence of surface chemistry on cycle capability in single-crystal battery materials. We, therefore, suggest that homogeneous phase distribution in single crystals during delithiation contributes to a negligible lithium-ion concentration gradients, which delivers small variations in stress distribution over all particles (Fig. 8e, f) and ensures excellent cycle performance. In this work, t-NCM tailored with a feasible method retains superior surface chemistry stability, contributing to homogeneous phase transformations observed in particles during long-term electrochemical cycling. As a result, the structural integrity of t-NCM was well preserved and excellent electrochemical performance could be achieved.

## Discussion

Understanding the fundamental role of surface chemistry in single-crystal structure stability can offer new insights into Ni-rich, single-crystal, cathode materials used for lithium-ion batteries. In this work, the structural evolution and degradation mechanisms of Ni-rich single-crystal NCM622 were studied. Within *operando* X-ray imaging, spectroscopic technologies, and diffraction methods, we found that local structures around Ni cations were more severely disordered during long-term cycling, and unambiguously demonstrate close correlations between surface chemical character, phase transformation, and structural stability in single-crystal battery particles. Pristine surface chemistry, accompanying phase heterogeneity, and induced stresses deteriorate structural integrity along with cycle performance. Surface chemistry regulation can induce homogeneous phase distribution across single crystals, contributing to the improved surface chemical stability and performance retention. Our studies have shed new light on microstructural and chemical evolution in single-crystal particles, and offer insights into particle-level degradation mechanisms, which guides the development of advanced single-crystal battery materials with improved electrochemical and safety characteristics.

## Methods

### Materials synthesis

Large single-crystal NCM were synthesized using co-precipitation methods^22,45^. Ni_0.6_Mn_0.2_Co_0.2_(OH)_2_ precursors: A co-precipitation method was used here to fabricate Ni_0.6_Mn_0.2_Co_0.2_(OH)_2_ precursors. First, nickel sulfate (99.8%, Alfa Aesar, 0.6 mol L^−1^), cobalt sulfate (99.8%, Alfa Aesar, 0.2 mol L^−1^), and manganese sulfate (99.8%, Alfa Aesar, 0.2 mol L^−1^) were mixed to obtain a uniform metal ion solution. Ammonia water, a complexing agent, was then used to perform a complexation reaction with the metal ions. The obtained solution was subsequently placed in a reaction kettle with NaOH solution, heated, and stirred to induce the co-precipitation reaction. Finally, the mixed solution was centrifuged and dried to obtain a nickel–cobalt–manganese precursor. Dried precursors obtained from the process steps were pre-calcined at 480 °C for 10 h.

#### NCM and t-NCM

single-crystal NCM materials were produced using high-temperature sintering. Mixing the NCM precursor with LiOH·H_2_O (Aladdin, 99.8%, Li: M ratio = 1.05:1), and the above mixture was then calcined at 950 °C for 10 h in pure oxygen to obtain single crystal NCM^41,42,46^. t-NCM material was prepared by a simple surface regulation method. First, the above obtained single-crystal NCM materials were mixed with extra LiOH using ball milling, and then the mixtures were calcined at 800 °C for 2 h under pure oxygen flow to obtain t-NCM.

### Electrode preparation and electrochemical measurement

Electrochemical tests were performed in the CR2025 coin-type cells. A slurry was prepared at room temperature by mixing of active material, conductivity agent (Super P), and binder (polyvinylidene fluoride) according to a weight ratio of 80:10:10, dissolved in N-methyl-1,2-pyrrolidone solution for 12 h. The slurry then was coated in a current collector (Al foil) and dried in a vacuum oven for 12 h at 80 °C. Active material mass loading in the electrodes was 2–3 mg cm^−2^. The coin-type cells were assembled including Li foil, composite cathode, and separator (Celgard 2025) using electrolyte (1 mol L^−1^ LiPF_6_, EC:EMC = 1:2 vol%) with 2 wt% vinylene carbonate as an additive in a glovebox filled with Ar.

Cycle performance and rate capacity were tested on a battery testing system (BTS-2004, Netware) ranging from 0.1 to 5 C between 2.8 and 4.7 V at room temperature (25 °C). Cyclic voltammetry was conducted on the CHI660E electrochemical workstation at 0.1 mV s^−1^ and 2.8−4.7 V potential. AC impedance (EIS) was performed in the PARSTAT 2273 instrument at a 5 mV amplitude and a frequency ranged from 0.1 MHz to 0.01 Hz.

#### X-ray absorption spectroscopy

Hard XAS measurements were performed on beamline 4-1 at the SLAC National Accelerator Laboratory (SLAC). Calibration was carried out using the first inflection point of the K-edge spectrum of the element foil (e.g., Ni, 8333 eV) as a reference. XANES and EXAFS spectra were resolved by the Athena software package. Soft XAS experiments were performed in TEY modes at beamline BL10B of the NSRL. Beam size was 1 mm in diameter.

#### Simultaneous X-ray fluorescence and ptychography

The combination of X-ray fluorescence and ptychography gives two complementary contrast modes of the sample: the fluorescence provides the distribution of many elements while XANES provides the chemical state of a single element of interest; whereas the ptychographic image reconstructed from diffraction patterns shows quantitative density map (or structure image) with a spatial resolution which can be much higher than the TXM. Ptychography-XANES has been demonstrated to obtain the chemical composition mapping on Li-ion battery particles with a state-of-the-art spatial resolution reaching sub-10 nm.

Measurements were performed on cycled NCM single crystal particles at 2-ID-D fluorescence microscope at the Advanced Photon Source (APS) in Argonne National Laboratory. An 8.8 keV monochromatic X-ray beam was focused by a Fresnel zone plate with an outermost zone width of 70 nm on to NCM particles. The focused X-ray probe with a diameter of about 150 nm was raster-fly-scanned across the sample, for every 60 nm sample motion a Vortex silicon drift detector (mounted at 90° to the X-ray beam direction)and a Dectris Eiger X 500 k hybrid pixel array detector (1.32 m downstream of the sample) were simultaneously triggered for 50 ms to record fluorescence spectra and coherent diffraction patterns, respectively^47^. At the end of the scan, those diffraction patterns were input into a GPU-based code to reconstruct high-resolution structure images of particles with a pixel size of 9.7 nm in real space^48^. The elemental fluorescence maps had a pixel size equal to the scan step size of 60 nm, however, their resolution was limited by the X-ray probe size which was about 150 nm.

#### 2D TXM

In *operando* 2D TXM-XANES experiments were performed on FXI beamline at NSLS-II. These electrodes were composed of active materials, carbon black and binder (4:4:2 in weight). In the construction of cell models, carbon papers and binders are necessary, but X-rays can transmit through these substances, so the authenticity of data will not be affected. To capture the correlation of phase change to the state of charge, the phase distribution of the battery in the 1st and 201st cycles were collected. The TXM at FXI beamline can obtain individual 2D projection images at 30 nm spatial resolution. A CCD camera with a field of view of 40 × 40 μm^2^ was employed, which means multiple particles can be observed simultaneously. The exposure times can be <50 ms per image, so one can catch the fast electrochemical reaction process. The 2D TXM-XANES images were collected at different states of charge by scanning Ni element K-edge ranging from 8313 to 8413 eV, with 1 eV step size, which generated 2k × 2k XANES spectra. In this work, each image was collected with 0.02 s exposure time^49^.

#### HAADF-STEM

Electron tomography and HAADF-STEM imagings were collected by a JEOL JEM-2100F operated at 200 kV. HAADF-STEM images and elemental mapping analysis were conducted on a Hitachi HD2700C. And these images were captured in sufficiently thin domains of the particles, owing to the resolution is limited by the thickness of the material.

#### Computational method

Interfacial models were constructed for randomly distributed, non-reactive regions, that contained homogeneous phase transformations within particles. Moreover, in the simulation of stress distribution the anisotropic deformation of NCM was considered, and the detailed tensor *D*_*ij*_ represents the diffusion coefficient. We set *D*_11_ = *D*_22_ = 1 × 10^−13^ m^2^ s^−1^, *D*_33_ = 1 × 10^−18^ m^2^ s^−1^, and *D*_*ij*_ = 0 for the other entries. The detailed calculation process is as follows. Chemical strain invoked by lithium extraction was assumed to be proportional to the normalized lithium concentration (c) at the fully lithiated state, as $\varepsilon _{ij}^c = \beta _{ij}c$. The diagonal tensor, *β*_*ij*_, represents the lithiation expansion coefficients. As for NCM622, we set *β*_11_ = *β*_22_ = 2.8%, *β*_33_ = −4.0%, and *β*_*ij*_ = 0 for the other entries^50^. Since it is an orthotropic crystal, the stiffness tensor of the layered structure depends on nine independent material constants. We set the material constants of NCM (*c* = 0) and LiNCM (*c* = 1) in the model, and assumed that the stiffness tensor of the intermediate stages scale linearly with lithium concentration^44^.

Diffusion induced stress calculations were performed using the finite element method with a commercial COMSOL Multiphysics package^41^. Diffusion induced stress caused by the insertion and extraction of Li^+^ was formulated following the thermal analogy. Constitutive equations describing the stress and strain are given by

$$\varepsilon _{ij} = \varepsilon _{ij}^e + \varepsilon _{ij}^c = \frac{1}{E}\left[ {\left( {1 + v} \right)\sigma _{ij} - v\sigma _{kk}\delta _{ij}} \right] + \beta _{ij}c,$$

$${\it{\upsigma }}_{\boldsymbol{r}} = \lambda {\it{e}} + {\mathrm{2}}{\it{\upmu \upvarepsilon }}_{\it{r}} - \frac{1}{3}\left( {3\lambda + {\mathrm{2}}{\it{\upmu }}} \right)\Omega \left( {{\it{C}} - {\it{C}}_{\mathrm{0}}} \right),$$

$$\sigma _\theta = \lambda e + {\mathrm{2}}{\it{\upmu \upvarepsilon }}_{\it{\theta }} - \frac{1}{3}\left( {{\mathrm{3}}{\it{\uplambda }} + {\mathrm{2}}{\it{\upmu }}} \right)\Omega \left( {{\it{C}}{\mathrm{ - }}{\it{C}}_{\mathrm{0}}} \right),$$

where *ɛ*_*ij*_ and *σ*_*ij*_ are the stretch tensor and stress tensor, respectively. *v* is Poisson’s ratio; *E* is Young’s modulus. For the diffusion problem, the transport of Lithium ions can be described by modified Fick’s law, including the effect of stresses on diffusion:

$$\frac{{\partial c}}{{\partial t}} + \nabla \cdot \left( { - D_{ij}\nabla c + \frac{{D_{ij}c}}{{RTc_{ref}}}\nabla {\sum} {\beta _{ij}\sigma _{ij}} } \right) = 0,$$

$${\it{\upmu }}_s = \upmu _0 + {\it{RT}}{\mathrm{ln}}{\it{C}} - \Omega \sigma _{\it{m}},$$

where *D*_*ij*_ is the diffusion coefficient tensor, and *R* is the universal gas constant. *T* is the temperature.

## Supplementary information

Supplementary Information

## Data Availability

The data supporting the findings of this study are available within the article and its Supplementary information files, or from the corresponding authors on reasonable request.
