# Supplementary material for: Surface regulation enables high stability of single-crystal lithium-ion cathodes at high voltage
Source: Nat Commun. 2020 Jun 16;11:3050. doi: 10.1038/s41467-020-16824-2 (PMC7298058; doi:10.1038/s41467-020-16824-2)
Supplement: Supplementary file 1 — Supplementary Information [file 41467_2020_16824_MOESM1_ESM.pdf]

Supplementary Information

Surface regulation enables high stability of single-crystal lithium-ion cathodes at high voltage

Zhang et al.

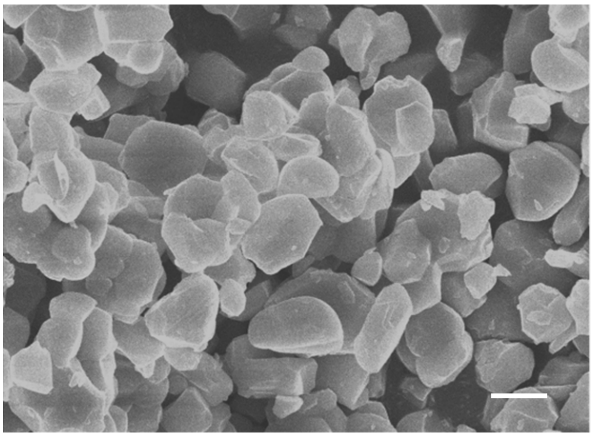

Supplementary Figure 1. **Morphological analysis of single-crystal NCM**. Low-resolution SEM image of NCM. Scale bar 2 µm.

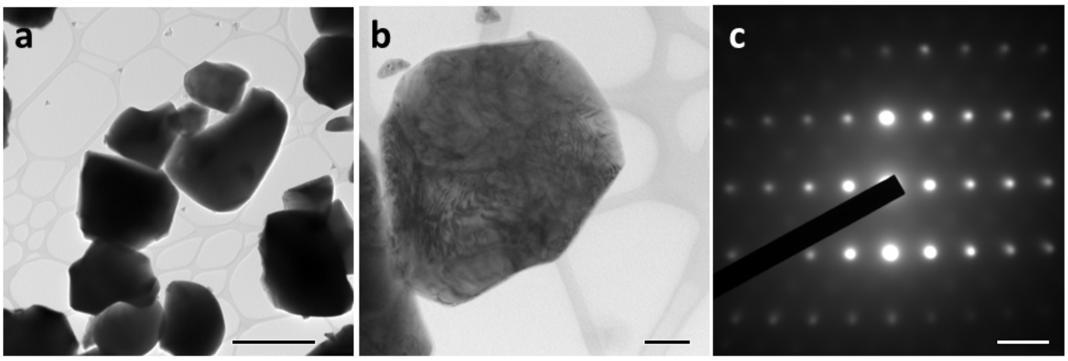

Supplementary Figure 2. **Morphological analysis of NCM**. **a** Low-resolution TEM BF images. Scale bar 2 µm. **b** and **c** BF-TEM image and the corresponding SAED pattern. Scale bar 200 nm, 5 nm.

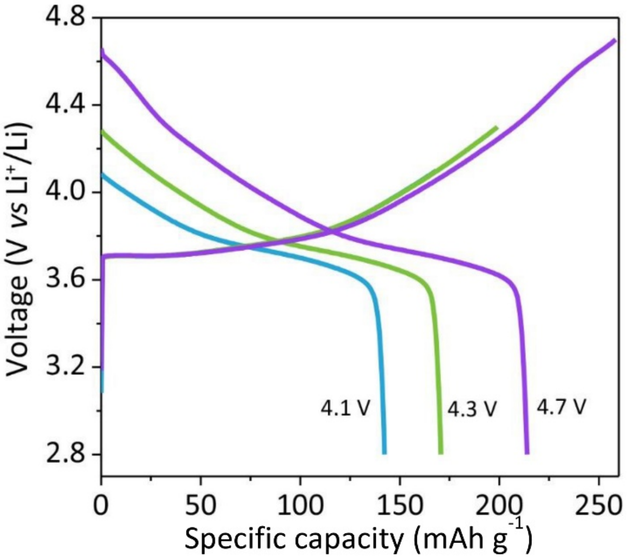

Supplementary Figure 3. Electrochemical performance of NCM. Initial charge-discharge profiles of NCM at 0.1 C under different cut-off voltages. (4.1 V, 4.3 V, 4.7 V).

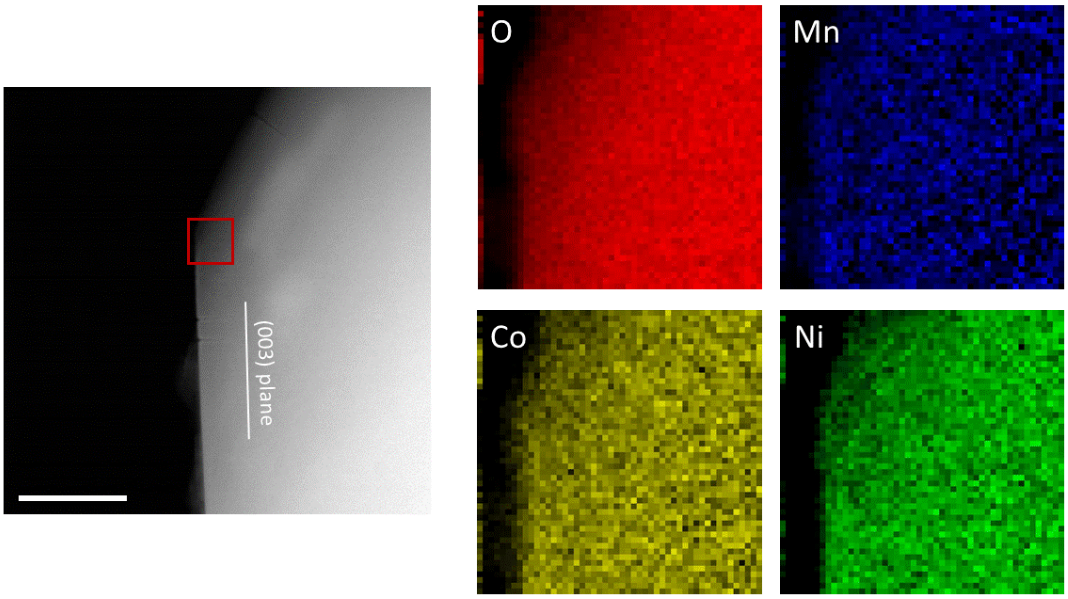

Supplementary Figure 4. Surface element analysis of NCM. HAADF-STEM-EDS mapping images of the pristine NCM. Scale bar 50 nm.

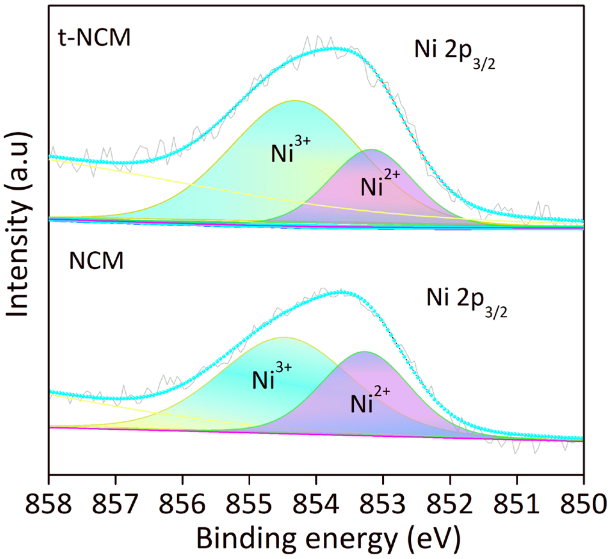

Supplementary Figure 5. Surface chemical analysis of pristine NCM and t-NCM. XPS profiles of pristine Ni 2p in NCM and t-NCM.

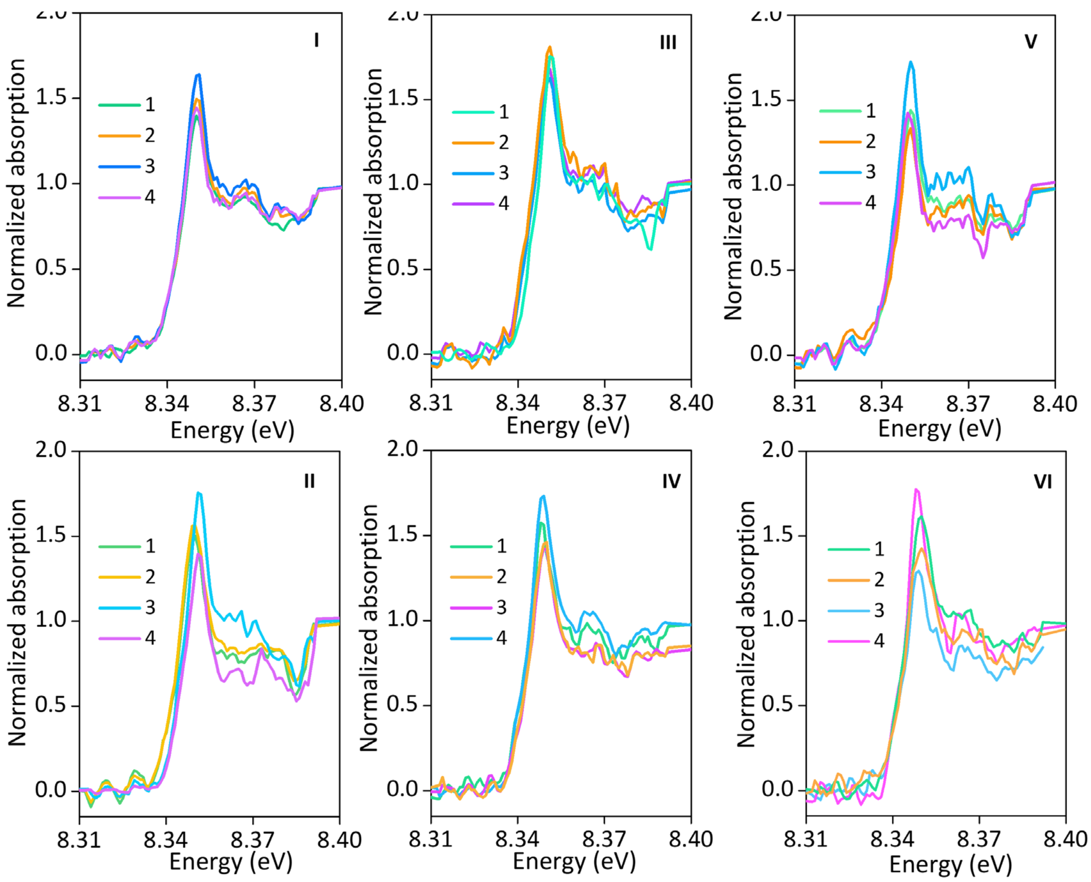

Supplementary Figure 6. Quantitative analysis of phase distribution. *Operando* 2D TXM-XANES (X-ray absorption near-edge structure) during the first and 201st cycle. 1st (I, pristine; III, 4.7 V; V, 3.6 V), and 201st cycle (II, pristine; IV, 4.7 V; VI, 3.6 V).

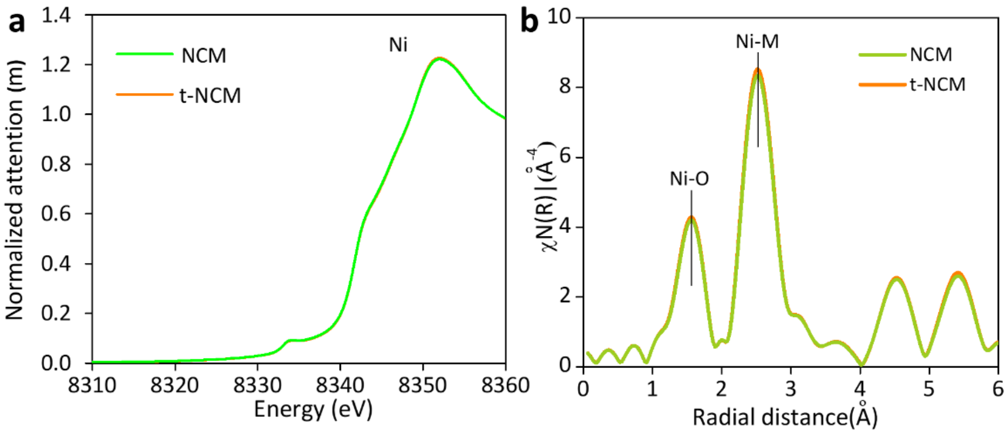

Supplementary Figure 7. Surface structure of NCM and t-NCM. a and **b** Ni K-edge XANES and EXAFS spectra of NCM and t-NCM crystals.

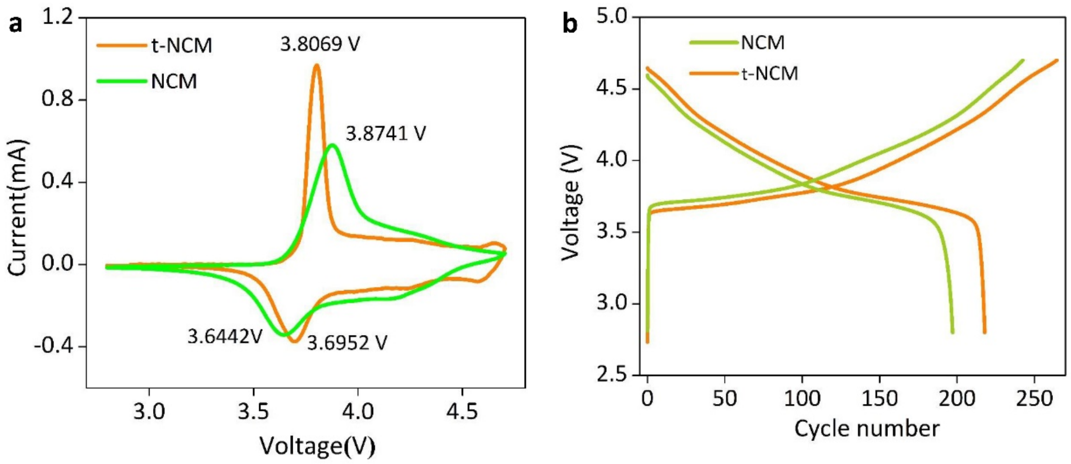

Supplementary Figure 8. First-cycle electrochemical performance. **a** Cyclic voltammograms at a scan rate of 0.1 mV s^{-1} for NCM and t-NCM. **b** Initial charge/discharge curves of NCM and t-NCM at 0.1 C.

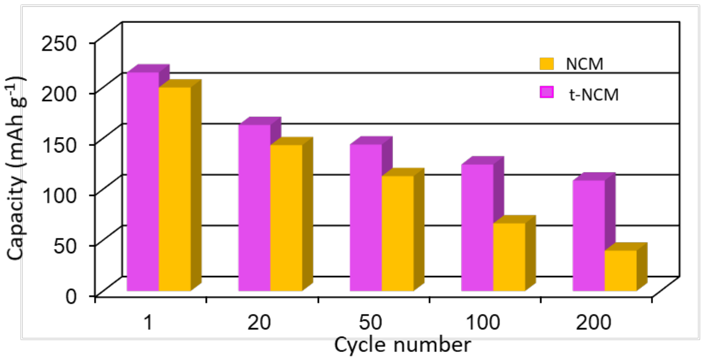

Supplementary Figure 9. Capacity performance of NCM and t-NCM. Discharge capacity of NCM and t-NCM after 1 cycle, 20 cycles, 50 cycles, 100 cycles, and 200 cycles.

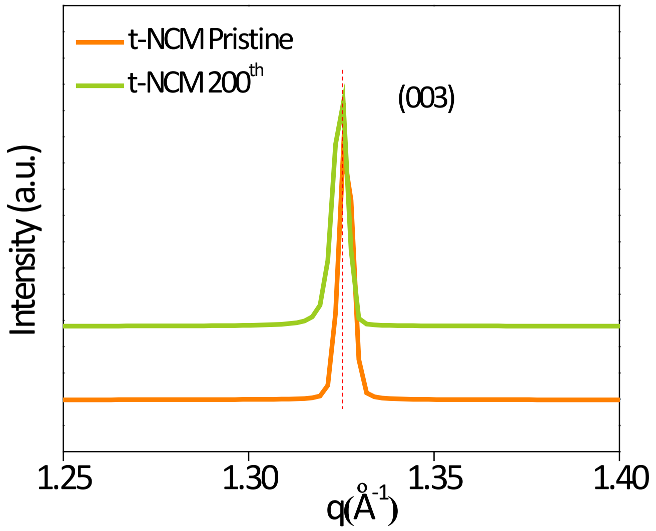

Supplementary Figure 10. Crystal structure stability of t-NCM. Synchrotron XRD signals for t-NCM samples. (003) peak shift for cycled sample).

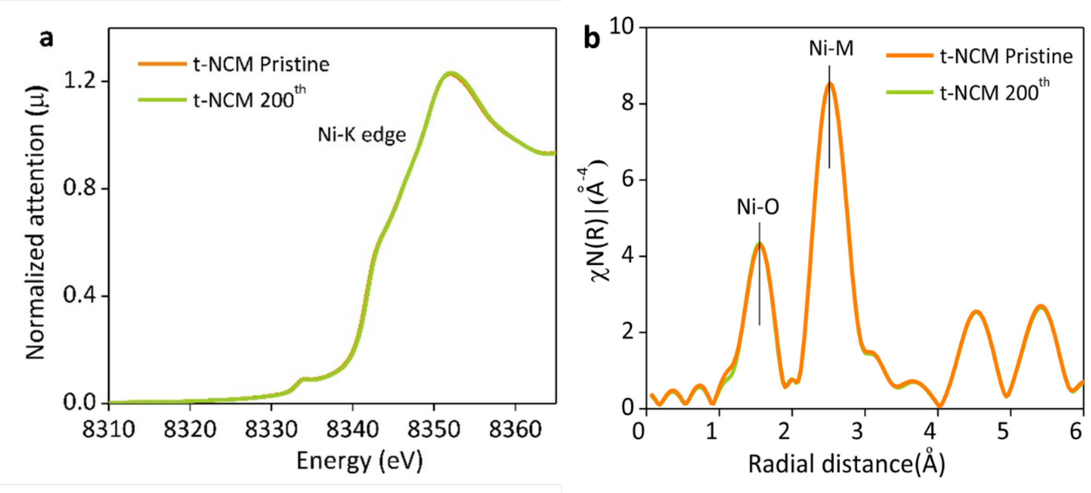

Supplementary Figure 11. Surface structure stability of t-NCM. a and **b** Ni K-edge XANES and EXAFS spectra of pristine and cycled t-NCM crystals.

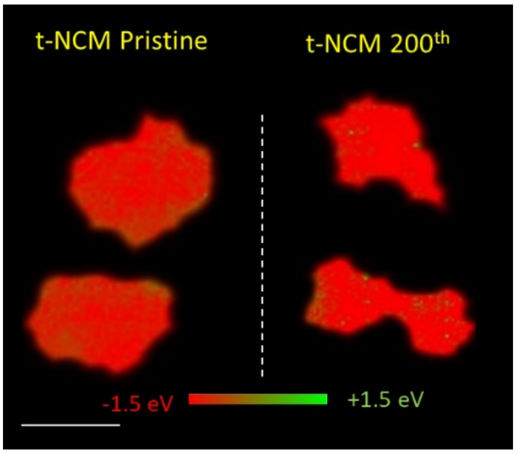

Supplementary Figure 12. Phase distribution analysis of pristine and cycled t-NCM. Ex-situ 2D TXM of pristine and cycled t-NCM. Scale bar 4 µm.

Supplementary Table 1. Atom coordinates and displacement parameters from NCM structure refinement results

NCM						
Site	Np	x	y	z	Atom	Occ
M	3a	0	0	0.5	Mn	0.1739
		0	0	0	Ni	0.6185
		0	0	0.5	Co	0.1739
		0	0	0	Li	0.8311
Li	3b	0	0	0.5	Li	0.0899
					Ni	0.0892
O	6c	0	0	0.2941	O	0.9831
	a=2.8676 Å<br>R_{wp}=2.5%			c=14.2077 Å		I(003)/I(104)<br>1.56

Supplementary Table 2. Atom coordinates and displacement parameters from t-NCM structure refinement results.

**t-NCM**						
Site	Np	x	y	z	Atom	Occ
M	3a	0	0	0.5	Mn	0.1902
		0	0	0	Ni	0.6521
		0	0	0.5	Co	0.1902
		0	0	0	Li	0.0693
Li	3b	0	0	0.5	Li	0.8497
					Ni	0.0692
O	6c	0	0	0.2941	O	0.9587
	a=2.8679 Å<br>R_{wp}=2.5%			c=14.2128 Å		I(003)/I(104)<br>1.79
